# The lateral supracerebellar infratentorial, translateral mesencephalic sulcus approach to the mesencephalopontine junction

**DOI:** 10.3171/2019.10.FocusVid.19454

**Published:** 2019-10-01

**Authors:** M. Yashar S. Kalani, Kaan Yağmurlu, Nikolay L. Martirosyan, Robert F. Spetzler

**Affiliations:** Department of Neurosurgery, Barrow Neurological Institute, St. Joseph’s Hospital and Medical Center, Phoenix, Arizona

**Keywords:** brainstem, cavernous malformation, mesencephalon, safe entry zone, surgery, video

## Abstract

The lateral supracerebellar infratentorial (SCIT) approach provides advantageous access to lesions located in the lateral mesencephalon and mesencephalopontine junction. For lesions that abut the pial surface, a direct approach is ideal and well tolerated. For deep-seated lesions, the lateral mesencephalic sulcus (LMS) can be used to access lesions with minimal morbidity to the patient. This video demonstrates the use of the SCIT approach via the LMS to remove a cavernous malformation at the level of the mesencephalopontine junction. The use of somatosensory and motor evoked potential monitoring and intraoperative neuronavigation is essential for optimizing patient outcomes. Meticulous, multilayered closure is critical for optimal results in the posterior fossa. For optimal patient outcomes, approach selection for deep-seated lesions should combine the two-point method with safe entry zones. At follow-up, the patient had persistent sensory changes but was otherwise neurologically intact.

The video can be found here: https://youtu.be/bHFEZhG8dHw.

## Transcript

This video illustrates the use of a lateral supracerebellar infratentorial craniotomy for resection of a cavernous malformation located in the pontomesencephalic junction. This 45-year-old female presented with atypical headaches. She had one episode of sudden-onset face and hand numbness 2 weeks prior to presentation. Imaging revealed a cavernous malformation located in the dorsolateral pontomesencephalic junction **(0:47)**. This anatomical dissection demonstrates the lateral view of the mesencephalon and the pontomesencephalic junction. Note the course of the fourth nerve and branches of the superior cerebellar artery over the lateral mesencephalic sulcus. The lateral mesencephalic sulcus has been described as a safe entry zone for resection of lesions located laterally at the level of the mesencephalon and upper pons. The fourth nerve can be traversing the sulcus **(1:18)**. For the lateral supracerebellar infratentorial craniotomy the patient is placed supine with the head turned to the contralateral side and the chin tucked. It is important to place the craniotomy at the junction of the transverse and sigmoid sinuses. This allows the surgeon to access the cerebellopontine angle to release cerebrospinal fluid to obtain cerebellar relaxation and then to use the supracerebellar approach to resect the lesion located in the brainstem. After releasing cerebrospinal fluid from the cerebellopontine angle cistern, the surgeon turns his attention supracerebellarly and develops this potential space between the cerebellum and tentorium. This allows the surgeon to arrive upon the lateral brainstem at the lateral mesencephalic sulcus, which has been described as a safe entry zone for entry into the pontomesencephalic junction **(2:10)**. An ideal point of entry into the brainstem is identified using the neuronavigation system. Note the course of the fourth nerve and SCA branches, which cross the lateral mesencephalic sulcus. A point should be selected that places the surgeon down the longest axis of the lesion being resected. A small opening in the brainstem is entered and developed large enough to place a suction and microdissector within the cavity. The use of the lighted suction allows for illumination at the depth of the surgical field **(2:45)**. The cavernous malformation is entered and resected in a piecemeal fashion, using a tugging technique to bluntly mobilize the cavernous malformation from adjacent structures. A dissector is used to develop the plain between the cavernous malformation and fibers of the mesencephalon. The cavernous malformation is then peeled out and resected. It is critical to obtain hemostasis at the depth of this long surgical field. The surgeon should be judicious about the use of cautery **(3:20)**. This final operative view demonstrates the orientation of the fourth nerve and the SCA relative to the side of the opening into the brainstem. Postoperative magnetic resonance imaging demonstrates complete removal of the lesion. The magnetic resonance imaging slides also demonstrate the trajectory used to enter the brainstem via the lateral mesencephalic sulcus safe entry zone.

## References

[1] Cavalcanti DD Preul MC Kalani MY Spetzler RF Microsurgical anatomy of safe entry zones to the brainstem J Neurosurg 124 1359 1376 2016 26452114 10.3171/2015.4.JNS141945

[2] Kalani MY Yagmurlu K Martirosyan NL Cavalcanti DD Spetzler RF Approach selection for intrinsic brainstem pathologies J Neurosurg 125 1596 1607 2016 27662530 10.3171/2016.6.JNS161043

[3] Spetzler RF Kalani MYS Nakaji P Yağmurlu K Color Atlas of Brainstem Surgery New York Thieme 2017

[4] Yagmurlu K Kalani MYS Preul MC Spetzler RF The superior fovea triangle approach: a novel safe entry zone to the brainstem J Neurosurg 127 1134 1138 2017 28009231 10.3171/2016.8.JNS16947

[5] Yagmurlu K Rhoton AL Jr Tanriover N Bennett JA Three-dimensional microsurgical anatomy and the safe entry zones of the brainstem Neurosurgery 10 Suppl 4 602 620 2014 24983443 10.1227/NEU.0000000000000466

